# Variations in urban land surface temperature intensity over four cities in different ecological zones

**DOI:** 10.1038/s41598-021-99693-z

**Published:** 2021-10-15

**Authors:** Ayansina Ayanlade, Michael I. Aigbiremolen, Olakunle R. Oladosu

**Affiliations:** 1 Department of Geography, Obafemi Awolowo University, Ile-Ife, Nigeria; 2 Cooperative Information Network (COPINE), Obafemi Awolowo University, Ile-Ife, Osun Nigeria; 3 Department of Geography and Regional Research, University of Vienna, Universitätsstraße 7/5, 1010 Vienna, Austria; 4 African Regional Centre for Space Science and Technology Education in English (ARCSSTE-E), Obafemi Awolowo University, Ile-Ife, Osun Nigeria

**Keywords:** Climate sciences, Environmental sciences, Environmental social sciences

## Abstract

This study aims at assessing variations and changes in the intensity of urban land surface temperature (LST) over four major cities in different ecological zone. The study intends to examine the contributions of different land cover types and variation in ecological locations on the intensity of urban LST. Remote Sensing and GIS techniques were used to measure the extent of the LST intensity over different cities and implications of land use/land cover (LULC) changes, using the Landsat TM/ ETM from 1984 to 2012, and Landsat OLI/TIRS from 2015 to 2019. The contributions of different landscape types to urban LST intensity were examined, using contribution index (*CI*) and Landscape index (*LI*) methods while the relationship between urban LST, and changes in LULC was examined using zonal statistics. The results revealed that the spatial and temporal changes in the LULC have greatly influenced the LST in the cities, though this varies from identified LULC. Changes in estimated LST vary from 0.12 to 1 °C yearly, while the changes are much intensified in the core section of the cities. The contribution of each landscapes varies, − 0.25 < *CI* > − 1.17 for sink landscape and 0.24 < *CI* > 1.05 for source landscape. The results further reveal that as *LI* ≥ 1, the contribution of source landscape to intensity of LST is lesser than that of sink landscape, but *LI* ≤ 1 shows that source landscapes contribute more to intensity of LST than sink landscapes. This might be as a result of changes in the vegetation cover between 1984 and 2019 as revealed in LULC change. Loss in the vegetal cover is anthropogenically induced leading to an increase in built-up and impervious surfaces resulted in mean monthly and yearly temperature changes. It is observed that the core and densities areas of cities witnessed higher LST compared with the rural area. The study concludes that different types of land cover within an urban area can affect the spatial pattern of urban LST, though this varies from one ecological zone to another and distribution of LST intensity in the urban area depends on its changes LULC. Thus, as cities’ population is expected to keep expanding there is a need to establish more viable linkages between the ever-growing population and land use patterns. The major findings from this study are useful in informing policymakers of the need to promote more sustainable urban development in the cities.

## Introduction

Change in urban land use is becoming noticeable in many cities, not only in developed countries but also in African cities, and this is most responsible for changes in urban land surface temperatures witnessed in recent years^1–5^. A combination of factors has been reported in the literature as the drivers of changes in LST. Such factors include the removal of vegetation within urban areas^6,7^, change in urban thermal and physical properties of construction materials, building, morphology, surface roughness^8,9^, and anthropogenic heat sources modify the local energy and urbanization, leading to increases in atmospheric temperature in urban areas compared to their surroundings^10–13^. Many of these studies concluded that the main causes of the recent intensification of LST in urban can be related to structural and land cover differences of urban and rural areas. Urban areas are rough with buildings extending above ground level and are dry and impervious with construction materials extending across natural soils and vegetation.

These urban characteristics alter the natural surface energy and radiation balances such that urban areas are relatively warm places^14,15^. Variations in the canopy-layer of urban heat occur close to the surface in cities and extend to approximately the mean building height. These changes have as well affected thermal, radiative, moisture and aerodynamic characteristics of the environment leading to the accumulation of heat in urban areas. Thus, a huge amount of vegetation cover in the cities is replaced by built-up surfaces, that absorbs incoming solar radiation and re-radiate it at night^16,17^. The urban canopy-layer is governed by local or micro-scale processes and refers to that portion of the planetary boundary layer whose characteristics are affected by an urban area^18,19^. Other studies have revealed that the LST difference is usually higher during the day than at the night^5,6,20^.

Different LST retrieval methods and platform data have been used in recent years though, the study of urban LST changes over the years have taken very varied dimensions, using both ground and satellite datasets. Satellite observations are used to study the changes in urban land surface temperature^21,22^. The remote sensing methods which have been used include the split-window method^14,23,24^, the single-channel algorithm^25,26^, temperature/emissivity separation method^27–29^, and mono-window algorithm^30–32^. Studies have assessed the performance of the mono-window algorithm (MWA), single-channel method (SCM), split-window algorithm (SWA-Q) and split-window algorithm (SWA-S). Although all of these methods can provide good results, the radioactive transfer equation works in conjunction with in situ measurements collected simultaneously with the satellite data, while the mono-window algorithm with radio sounding data can get a better result than the single-channel algorithm with a root mean square deviation of 0.9 K^33^, while Tsou et al.^34^ further reported that LST retrieval by SWA-Q had better accuracy than the other algorithms. Generally, what is obvious in literature is that the cities’ population is expected to keep expanding thus there is a need to establish more viable linkages between the ever-growing population and land use patterns, essentially because these are the factors directly related to the cities’ land surface temperature regime. On the other hand, raised temperatures from urban heat islands, especially throughout the midyear, can influence a community’s environment and the quality of life of people. Meanwhile, a steady and perceptible detailed study to assess the changes and effects of change in land cover on land surface temperature in cities across different ecological zones especially is rare. The present study, therefore, seeks to contribute to this by looking at past issues related to urban heat islands in the cities in different ecological zone in the tropics. The examination of variations in the cities’ land surface temperature, the establishment of the causes and effects of these variations, and the examination of the relationship between land surface temperature and land use land cover in the cities in the specified study period are thus essential. Hence, the present study aims at assessing land surface temperature in four selected cities, using satellite data. The specific objectives of the study are to examine variations in land surface temperature in the cities; and examine the relationship between land use/land cover change and land surface temperature.

## Methodology

### The study area

**Figure 1:**
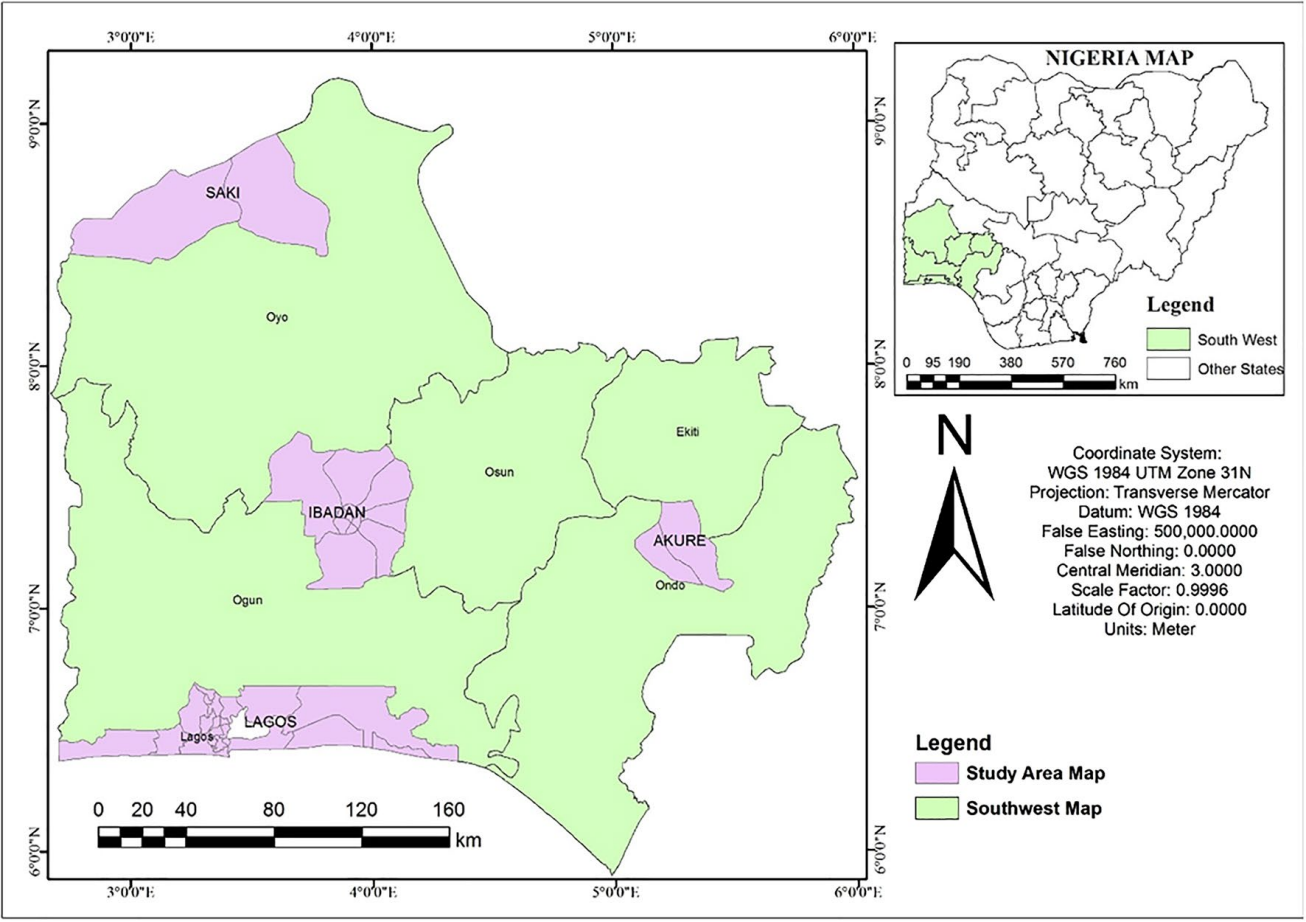
Map of the study area showing the location of four cities examined in the study. The figure was generated using ArcGIS 10.5 software (http://www.esri.com/).

This study involves different cities from ecological zones in Nigeria: Akure, Ibadan, Lagos, and Saki (Fig. 1). The study cities were purposively selected to cover different ecological zones: Guinean Savannah, derived Savannah, tropical rainforest and mangrove zones (Table 1). The four cities being considered in this study have experienced some level of urban growth over the years. In recent years, there has been a major increase in the built-up of the area in Akure but the thick forest vegetation has been massively depleted to give room for farming and other agricultural activities, a similar change in the land use from thick vegetal cover to agricultural farmlands could also be observed in Saki. Lagos is a large port city in southwestern Nigeria and one of the most rapidly developing cities in Africa with a landmass of about 2341.72 km^2^. Lagos is also a key cultural centre of the whole region, with very interesting and old local traditions, cuisine, music, and lifestyle. Tourism plays an important role in the city's economy, and most of the guests come to the city when it is time for numerous music festivals and cultural events. Ibadan city, the administrative capital of Oyo state, lies completely within the boundary between the tropical forest zone and derived savannah. According to the Koppen climate classification, it has a tropical wet and dry climate. Its mean total rainfall is 1420.06 mm. Its mean maximum temperature is 26.46 °C while its mean minimum temperature is 21.42 °C.

**Table 1:** Ecological characteristics of the cities examined in the study.

Ecological zones	Cities	Ecological characteristic
Tropical rainforest	Akure	Renowned for very high annual rainfall with high average temperatures, the rays of the sun are obstructed by the dense green canopy. The climate is generally hot and humid all year round, with continuous rain
Derived Savanna	Ibadan	The ecological zones are the result of people clearing forest land for cultivation. High average temperatures and periodic rainfall
Mangrove zones	Lagos	This ecology is located along the coast. It is also characterized by such qualities as a humid climate, saline waterlogged ecological region
Guinean Savanna	Saki	Prominent for relatively low rainfall which supports the growth of short deciduous trees

### Multi-year dataset acquisition

A multi-year Landsat dataset, from 1984 to 2019 were used in the study, to examine long period LST. The dataset cover both dry and wet seasons, at least a Landsat scene for a month, depending on low cloud coverage. The Landsat Thematic Mapper (TM), Enhanced Thematic Mapper Plus (ETM+) and an Operational Land Imager-Thermal Infrared Sensor (OLI-TIRS) dataset from 1984 to 2019 were used in this study to assess change in urban LST. The complete multi-year Landsat dataset was used to develop long term LST database for the four cities, results of 1984, 1994, 2004 and 2019 were presented to show the decadal variation. To examine the changes in Land use/Land cover (LULC) within and around the cities, as it varies over years, Landsat datasets for years 1984, 2004 and 2019 were used for classification.

### Land surface temperature retrieve and assessment

The LST over the cities were retrieved from the thermal bands of multi-year Landsat datasets. Thermal bands 6 (10.40–12.50 μm), 6H (10.4–12.5 μm) and 10 (10.60–11.19 μm) of TM, ETM + and OLI-TIRS respectively, with spatial resolutions of 120 m, 60 m and 100 m respectively were used in the study to distinguish nature urban surface temperature in different cities. Also, multi-year Landsat datasets as it relates to changes in land use and land cover (LULC) and NDVI, over four cities in southwestern Nigeria (Fig. 1) were acquired from the United States Geological Survey (USGS), using a path of 191 and a row of 54 for Lagos, path of 191and a row of 55 for Ibadan, the path of 190 and a row of 55 for Akure and path of 191 and a row of 55 for Saki (Table 2).

**Table 2:** Characteristics of the satellite dataset used in the study.

Satellite sensors/imagery characteristics	Thematic mapper (TM)	Enhanced thematic mapper plus (ETM +)	Landsat 8 (OLI & TIRS)
Bands	1 (0.45–0.52 μm) 2 (0.52–0.60 μm) 3 (0.63–0.69 μm) 4 (0.76–0.90 μm) 5 (1.55–1.75 μm) 6 (10.4–12.5 μm) 7 (2.08–2.35 μm)	1 (0.45–0.52 μm) 2 (0.52–0.60 μm) 3 (0.63–0.69 μm) 4 (0.76–0.90 μm) 5 (1.55–1.75 μm) 6 (10.4–12.5 μm) 7 (2.08–2.35 μm) panchromatic band 8 (0.50–0.90 μm)	1 (0.45–0.52 μm) 2 (0.52–0.60 μm) 3 (0.63–0.69 μm) 4 (0.76–0.90 μm) 5 (1.55–1.75 μm) 6 (10.4–12.5 μm) 7 (2.08–2.35 μm) panchromatic band 8 (0.50–0.90 μm)
Spatial resolution	30 m (bands 1–5, 7) 120 m (band 6)	30 m (bands 1–5, 7) 60 m (band 6) 15 m/18 m (band 8)	28.5
Radiometric resolution	8 bits	best 8 of 9 bits	16 bits
Path and row	191/055 191/054 190/055	191/055 191/054 190/055	191/055 191/054 190/055
Pre-processing history	Geometric corrected Terrain corrected	Geometric corrected Terrain corrected	Geometric corrected Terrain corrected

The datasets were acquired in December 2019.

Subsequently resampling the spatial resolutions to a uniform resolution of 30 m, these thermal bands were used to retrieve LST over the study area for three different periods were done following five major steps: (1) converting Digital Numbers (DNs) to spectral radiance, (2) obtaining effective at-sensor brightness temperature also known as black body temperature from the spectral radiance using Plank’s inverse function, (3) estimating land surface emissivity, (4) extracting urban land surface temperature, and (5) converting the final LST values to degrees Celsius^5,22,35^. Conversion of Digital Numbers (DNs) to spectral radiance was performed using the equation:

1 $${\text{L}}_{\lambda } = \left( {{\text{M}}_{{\text{L}}} \times {\text{Q}}_{{{\text{Cal}}}} } \right) + {\text{A}}_{{\text{L}}}$$

where *L*_*λ*_ is the spectral radiance at the sensor’s aperture in Wm^−2^ sr^−1^ μm^−1^; Q _Cal_ is quantized calibrated pixel values in *DN*s, and *M*_*L*_ and *A*_*L*_ are band-specific rescaling factors given in the metadata file as radiance multi-band *x* and radiance add band *x* respectively.

The effective at-sensor brightness temperature also known as black body temperature (*T*_*B*_) was obtained from the spectral radiance using Plank’s inverse function:

2 $$T= \frac{{K}_{2}}{In \left(\frac{{\in K}_{1}}{{L}_{\lambda}}\right)+ 1}$$

where *T*_*k*_ is at-satellite brightness temperature (in Kelvin); *K*_*2*_ is a calibration constant in Wm^−2^ sr^−1^ μm^−1^; K_1_ is calibration constant 1 in Wm^−2^ sr^−1^ μm^−1^; and *L*_*λ*_ is the spectral radiance at the sensor in Wm^−2^ sr^−1^ μm^−1^. However, the land surface emissivity estimation was calculated using the temperature values obtained using Eq. (2), which referenced to a blackbody. Therefore, corrections for spectral emissivity (ε) are necessary according to the nature of the land cover.

### Valuation of contributions of different landscapes to urban LST intensity

The contributions of different landscape types to urban LST intensity were examined, using the contribution index (*CI*) and Landscape index (*LI*) approach. The *CI* was used to examine both the spatial and temporal distinctive characteristics of both vegetation (sink) and non-vegetation (source) landscapes and how they contribute to the development/changes of urban LST. The urban landscapes were grouped into two: sink and source landscapes. The source landscape types are characterized by built-up surfaces part of the cities which are distinguished with the concrete surface while sink landscapes are mainly vegetation and water surfaces. The contribution of both sink and source landscapes to urban LST intensity were calculated *CI* as in Eq. (3):

3 $$CI= {D}_{t} \times {S}$$

*Dt* represents the difference in the temperature between the sink or source landscape and the entire region while the proportion of the area that is source landscape or sinks landscape in the entire area are present as *S*. Thus for sink landscape, the contribution to LST intensity is the fraction of the area (*S*) that is part of the sink landscape multiplied by the difference in temperature (*Dt*) between the sink landscape and the entire region. For a sink landscape, the *Dt* values are negative but the contribution index is also positive for the source landscape. The landscape index approach was used to determine the contribution of both landscapes to the urban LST intensity as in Eq. (4):

4 $$LI=\left|{CI}_{sink}/{Ci}_{source}\right|$$

where landscape index (*LI*) is the function of the contribution index (*CI*) of both source and sink landscapes. This evaluated how the changes in land use/land cover within and around the cities affect the intensity of urban LST. Also, zonal statistics were used to calculate the relationship of the statistics and to show if a landscape zone feature contains overlapping the LST zones. This was also used to compare the intensity of the LST in a different zone.

### Changes in land use/land cover as relate to urban LST intensity

Multispectral image classification was used to extract thematic information from satellite images. It aids in having a good understanding of the different land cover classes found within an area of interest. Classification in this study was performed using the Maximum likelihood classification form. The maximum likelihood method of classification algorithm is one of the common parametric classifiers used for supervised classification^36,37^. The algorithm is used for Computing the weighted distance or likelihood (*D*) of unknown measurement vector (*X*) belonging to one of the known classes (*Mc*) which is based on the Bayesian equation:

5 $${\text{D}} = {\text{ln}}\left( {{\text{ac}}} \right) - \left[ {0.{\text{5ln}}\left( {{\text{covc}}} \right)} \right] - \left[ {0.{5}\left( {{\text{X}} - {\text{Mc}}} \right){\text{ T}}\left( {{\text{covc}} - {1}} \right) \, \left( {{\text{X}} - {\text{Mc}}} \right)} \right]$$

A supervised classification was performed by creating a training sample, and based on a spectral signature curve, different land-use classes were created including Built-up area, Waterbody, Cultivation, Natural vegetation and Rock outcrop. While the land surface temperature was extracted using the equation:

6 $${\text{LST}} = {\text{T}}_{{\text{B}}} /{1} + \left( {\lambda \times {\text{T}}_{{\text{B}}} /\rho } \right) \times {\text{ln}}\varepsilon$$

Since the LST values were obtained in degree Kelvin, LST was converted into degrees Celsius. The LST is the land surface temperature is the function of *T*_*B,*_ λ, ρ and ε, such as λ is the central wavelength of emitted radiance (11.45 μm for TM and ETM+; 10.90 μm for OLI-TIRS); *T*_*B*_ is at-satellite brightness temperature; ρ is h × c/σ (1.438 × 10^−2^ mK or 14,380 µmK); h is Planck’s constant (6.26 × 10^−34^ Js); c is the velocity of light (2.998 × 10^8^ m/s); σ is Stefan Boltzmann’s constant (1.38 × 10^−23^ J K^−1^); and ε is the land surface emissivity. In this study, the relationship between LST and vegetation availability was calculated using zonal statistics.

Likewise, each of the LULC categories is assigned an emissivity value by reference to the emissivity classification scheme by Snyder et al.^38^. A heterogeneous surface (that is, a mixture of bare soil, exposed water surface and vegetation) was considered in this study. The emissivity of a heterogeneous surface (mixed pixels) was computed using Eq. (3), taking into account the proportion of vegetation in each pixel (*f*_*v*_) and the cavity effect due to surface roughness (C_λ_).f_v_ was estimated using Eq. (4)^33^, while *C*_*λ*_ was calculated using Eq. (5), with the geometrical (shape) factor ‘F’ having the mean value 0.55, according to Sobrino et al.^39^:

7 $$\varepsilon = \varepsilon_{{\text{v}}} \times {\text{f}}_{{\text{v}}} + \varepsilon_{{\text{s}}} \times \left( {{1} - {\text{f}}_{{\text{v}}} } \right) + {\text{C}}_{\lambda }$$

8 $${\text{f}}_{{\text{v}}} = \left[ {{\text{NDVI}} - {\text{NDVI}}_{{\text{s}}} /{\text{NDVI}}_{{\text{v}}} - {\text{NDVI}}_{{\text{s}}} } \right]^{{2}}$$

9 $${\text{C}}_{\lambda } = \left( {{1} - \varepsilon_{{\text{s}}} } \right) \times \varepsilon_{{\text{v}}} \times {\text{F}}\prime \times \left( {{1} - {\text{f}}_{{\text{v}}} } \right)$$

NDVI is the Normalized Difference Vegetation Index as computed in Eq. (10) for the respective years considered in this study;

10 $${\text{NDVI}} = {\text{NIR}}{-}{\text{RED}}/{\text{NIR}} + {\text{RED}}$$

*NDVI*_*s*_ and *NDVI*_*v*_ are Normalized Difference Vegetation Index Threshold values for soil pixels (*NDVI*_*s*_ = 0.2) and pixels of full vegetation (*NDVI*_*v*_ = 0.5) respectively (Eq. 4), as proposed by Sobrino et al.^39^ and Sobrino et al.^33^; and ε_s_ and ε_v_ are the emissivity (ε) of soil pixels and full vegetation pixels with the mean values 0.97 and 0.99, respectively. The final expression for land surface emissivity is given by:

11 $$\varepsilon \, = 0.00{\text{4f}}_{{\text{v}}} + 0.{986}$$

**Figure 2:**
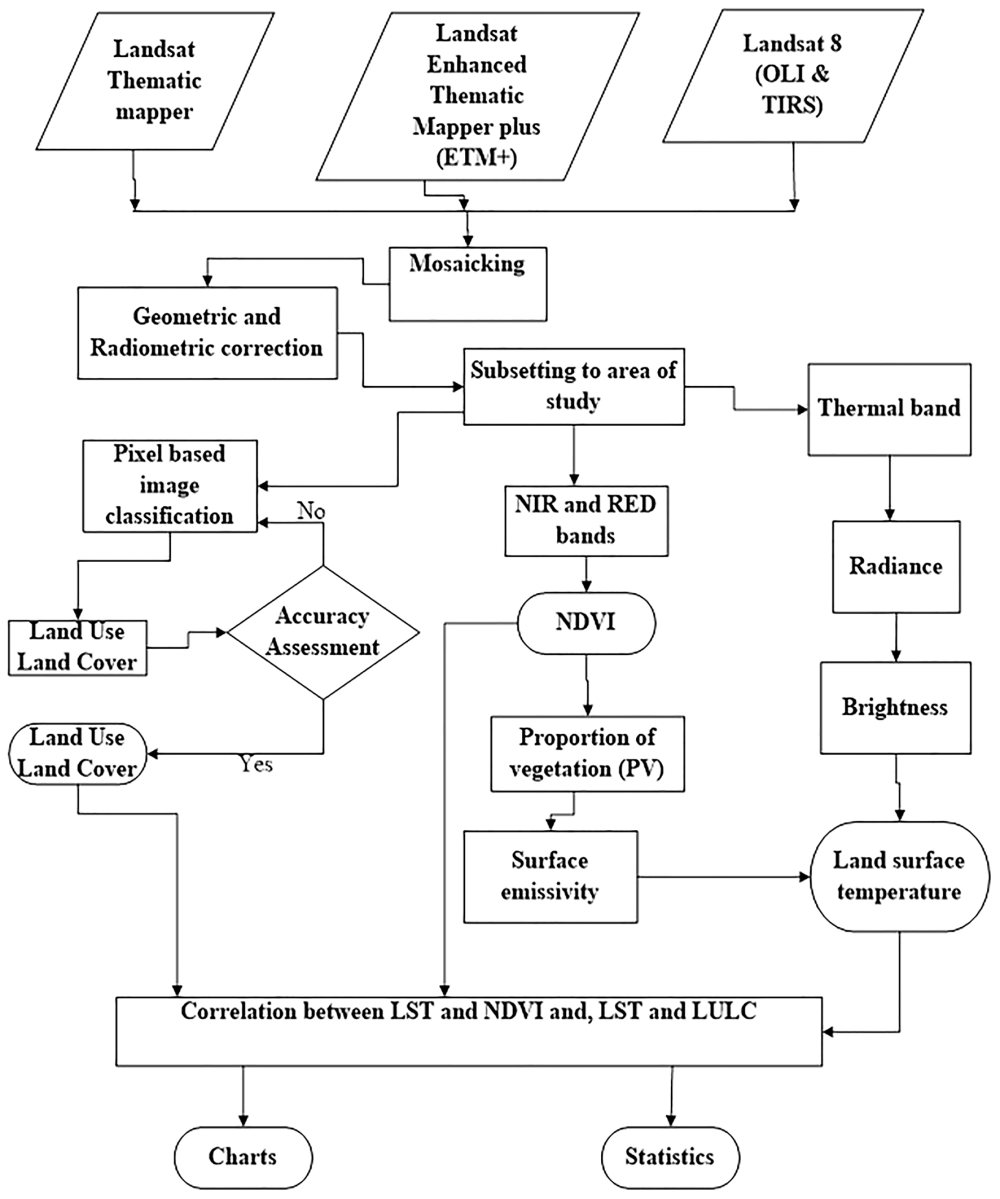
Flow chart showing the detailed steps of methods and material use in the study. This figure was designed by Michael I Aigbiremolen using flowchart symbols in Microsoft Word.

Detail flow of the methodology is presented in Fig. 2.

## Results and discussion

In this study, the results revealed three major outcomes: (a) the cities centres are much warmer and this increased considerably over the years; (b) the contribution index (CI) of source landscapes are positive while the values are negative for sink landscapes in all cities irrespective of their ecological location; (c) changes in land use/land cover within and around the cities affect the intensity of urban LST.

### Contribution of different landscapes to LST intensity

Different landscapes contribute differently to the intensity of LST. The contribution indexes (*CI*) of source landscapes are positive while the values are negative for sink landscapes (Tables 3, 4, 5, 6). The size (*S*) of both landscapes changes (Tables 3, 4, 5, 6) over the years, as source landscapes increases, the sink landscapes decreases from 1984 to 2019. The *S* values for sink and source landscapes, in Akure for example, changes from were 67% and 33% in 1984 to 49% and 51% in 2019, respectively. Similar increases were observed in all other cities but much more high in Ibadan and Lagos. In Ibadan, the *S* value for sink landscape was 40% in 1984 but changed to 29% in 2019 while source landscape change changed from 60% in 1984 to 71% in 2019 (Table 5). Much of these changes are observed in Lagos where *S* value for sink landscape was 47% in 1984 but changed to 25% in 2019 while the source landscape change changed from 53% in 1984 to 75% in 2019. Both spatial and temporal changes in the landscapes types affect the *CI* of each landscape and the intensity of the LST in all the cities (Tables 3, 4, 5, 6). In terms of the individual contribution of each landscape type in the cities of study, source landscape contributes positively to the intensity of LST while sink landscape has a negative contribution as presented in *CI* in Tables 3, 4, 5, and 6. The range of contribution varies, − 0.25 < *CI* > − 1.17 for sink landscape and 0.24 < *CI* > 1.05 for source landscape. The contributions of source landscapes are much more intense in recent years, especially in Lagos with *CI* = 1.05 in 2004 and *CI* = 0.82 in 2019. These results imply that the source landscape contributes mostly to the intensity of the urban LST in the study cities.

**Table 3:** Variations in LST of the source and sink with their landscape index over Akure.

Year	Landscape	*S*	*Dt*	*CI*	*LI*
1984	Sink	67	− 0.935	− 0.626	1.834
	Source	33	1.035	0.342	
1994	Sink	63	− 0.735	− 0.463	1.368
	Source	37	0.915	0.339	
2004	Sink	58	− 0.515	− 0.299	0.777
	Source	42	0.915	0.384	
2019	Sink	49	− 1.015	− 0.497	0.569
	Source	51	1.715	0.875

**Table 4:** Variations in LST of the source and sink with their landscape index over Ibadan.

Year	Landscape	*S*	*Dt*	*CI*	*LI*
1984	Sink	40	− 0.986	− 0.394	1.584
	Source	60	0.415	0.249	
1994	Sink	38	− 0.819	− 0.311	1.057
	Source	62	0.475	0.294	
2004	Sink	31	− 0.975	− 0.302	0.922
	Source	69	0.475	0.328	
2019	Sink	29	− 0.475	− 0.138	0.408
	Source	71	0.475	0.337

**Table 5:** Variations in LST of the source and sink with their landscape index over Lagos.

Year	Landscape	*S*	*Dt*	*CI*	*LI*
1984	Sink	47	− 1.390	− 0.653	1.131
	Source	53	1.090	0.578	
1994	Sink	37	− 1.495	− 0.553	1.496
	Source	63	0.587	0.370	
2004	Sink	30	− 1.495	− 0.449	0.429
	Source	70	1.495	1.047	
2019	Sink	25	− 1.195	− 0.299	0.364
	Source	75	1.095	0.821

**Table 6:** Variations in LST of the source and sink with their landscape index over Saki.

Year	Landscape	*S*	*Dt*	*CI*	*LI*
1984	Sink	68	− 1.715	− 1.166	1.895
	Source	32	1.923	0.615	
1994	Sink	58	− 1.734	− 1.006	1.319
	Source	42	1.815	0.762	
2004	Sink	58	− 1.523	− 0.883	1.253
	Source	42	1.678	0.705	
2019	Sink	45	− 1.345	− 0.605	0.677
	Source	55	1.625	0.894	

**Figure 3:**
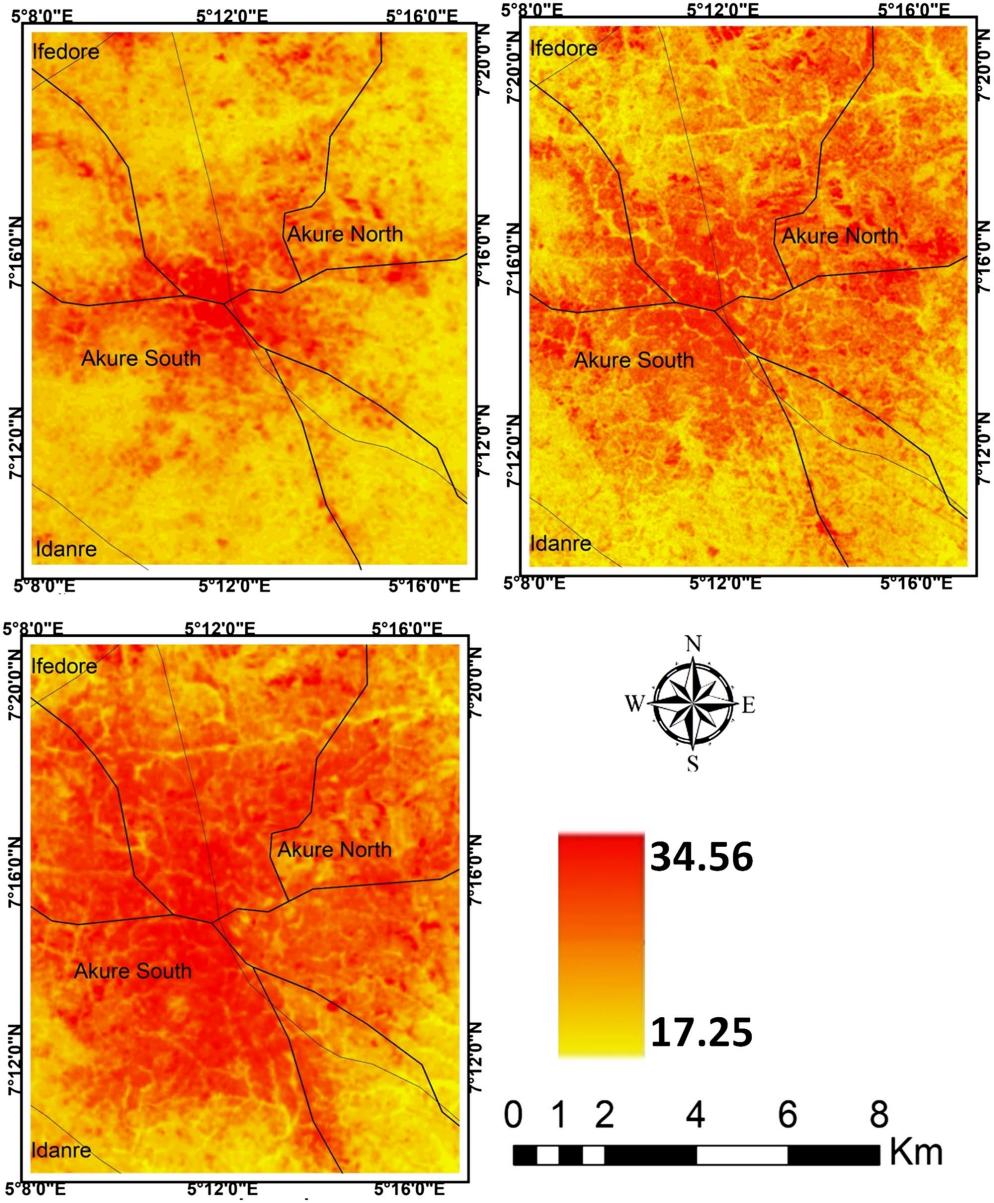
Land surface temperature intensity over Akure from 1984 to 2019. The figure was generated using Quantum GIS (QGIS 3.4) software (https://qgis.org/en/site/).

**Figure 4:**
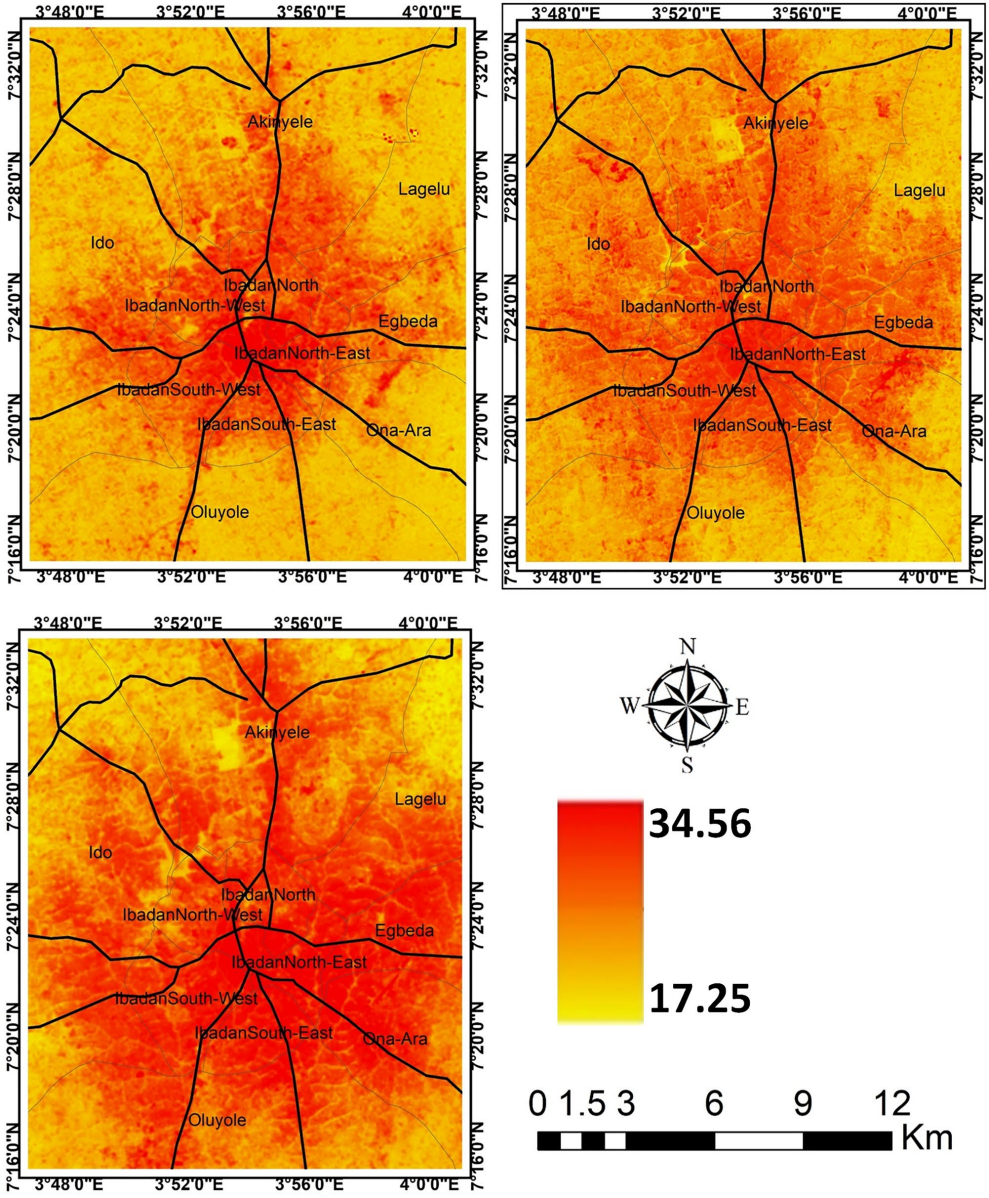
Land surface temperature intensity over Ibadan from 1984 to 2019. The figure was generated using Quantum GIS (QGIS 3.4) software (https://qgis.org/en/site/).

**Figure 5:**
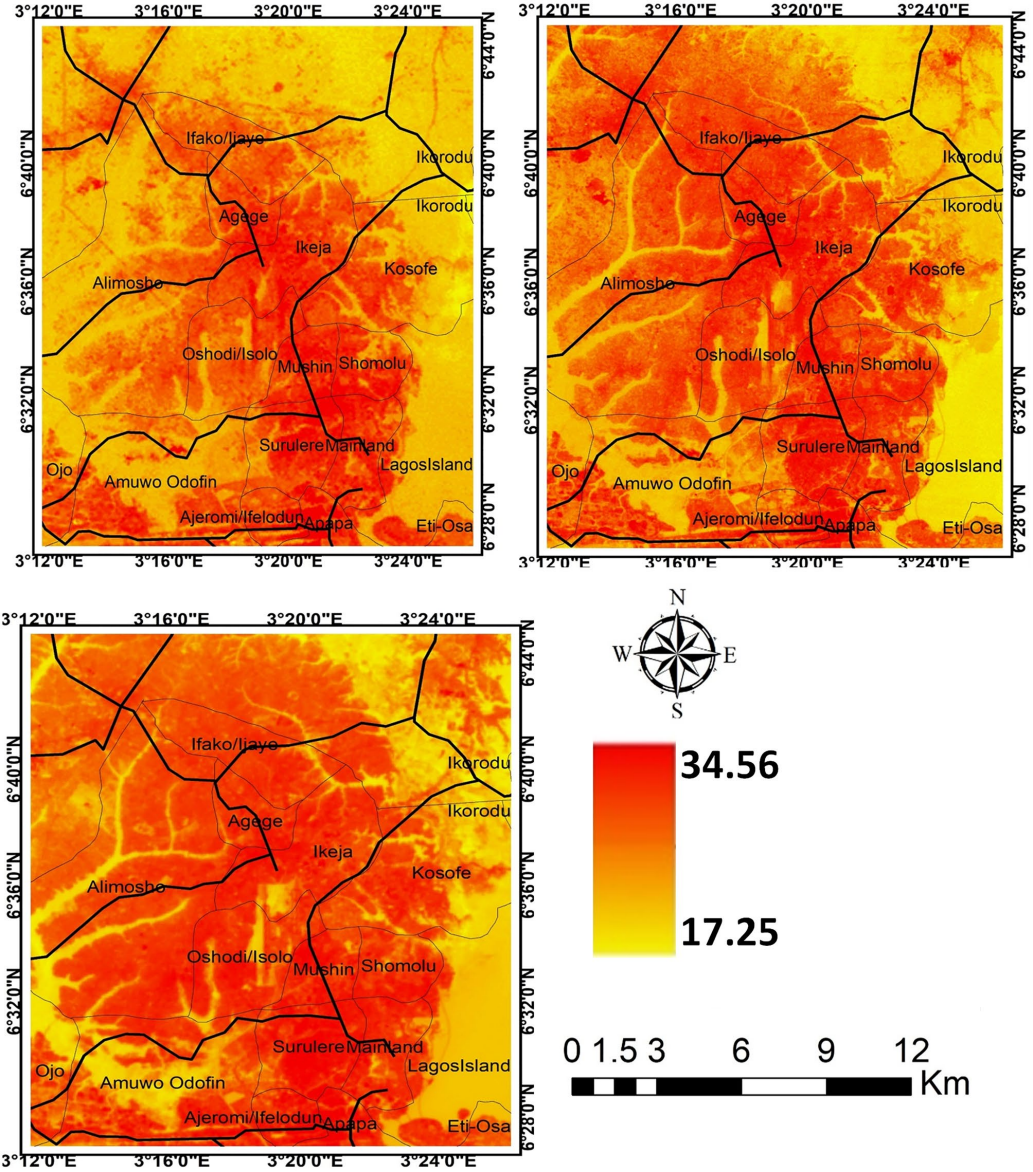
Land surface temperature intensity over Lagos from 1984 to 2018. The figure was generated using Quantum GIS (QGIS 3.4) software (https://qgis.org/en/site/).

**Figure 6:**
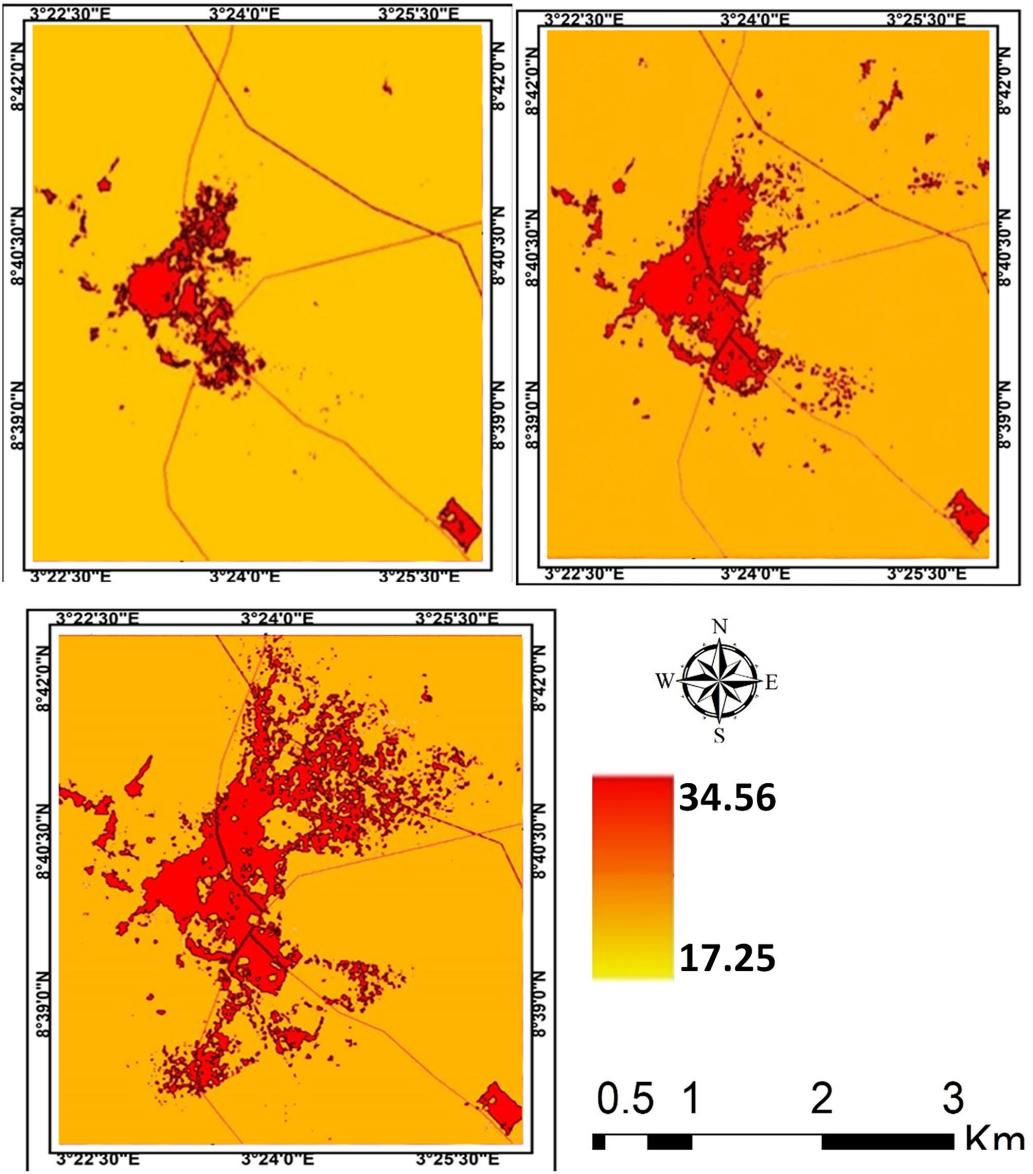
Land surface temperature intensity over Saki from 1984 to 2019. The figure was generated using Quantum GIS (QGIS 3.4) software (https://qgis.org/en/site/).

The result of *LI* further revealed that the contribution of the landscape types to the intensity of LST in the cities varies. In all the cities, it is obvious that *LI* decreases from *LI* = 1.84 in 1984 to *LI* = 0.57 in 2019 for Akure (Table 3); from *LI* = 1.58 in 1984 to *LI* = 0.41 in 2019 for Ibadan(Table 4); from *LI* = 1.13 in 1984 to *LI* = 0.36 in 2019 for Lagos (Table 5); and from *LI* = 1.90 in 1984 to *LI* = 0.677 in 2019 for Saki (Table 6). The results imply that as *LI* ≥ 1, the contribution of source landscape to intensity of LST is lesser than that of sink landscape, but *LI* ≤ 1 shows that source landscapes contribute more to intensity of LST than sink landscapes (Tables 3, 4, 5, 6). In all the cities examined in this study, the core part of each city was much warmer compared to the neighbouring areas (Figs. 3, 4, 5, 6). The intensity of the LST increased over the years but much more in the centre of the cities.

The general observation in Akure is that only the central part of the city was much warmer in 1984 compared to other past of the city but a significant increase in LST was observed towards the northeastern part of Akure as of 2004 and this scenario appears much more towards the northwestern and southern part of the city in 2019 (Fig. 4).

The intensity of LST clusters in high-density built-up areas in the different cities. It implies thus that the expansion of built-up areas further influences the intensity of LST in the urban area. The intensity of LST increases along the region of the cities with the high rate of high-density and expansion of built-up areas. These results further indicate more of a distinction between the built-up and the vegetal cover in the area as they contribute to the intensity of LST in the study cities. However, the observation of temperature that show a higher mean temperature in Saki compared to other cities. The ecological location of Saki, in Guinean Savannah, may be responsible for this. The intensity of LST increases from year to year as the density of built-up areas expanding. In Lagos for example, it could be visually seen (Fig. 5) high cluster of LST in the southern part of the city in 1984 but there appears a significant spread with much more intensity of LST across every part of the city as observed in 2019. In other cities, the high cluster of LST is obvious in the later years from 2004 to 2019. The reasons for these outcomes are very obvious as the majority of source landscapes in the cities centres are usually built-up surfaces distinguished with the concrete surface while sink landscapes are mainly vegetation and water surfaces. This is much more apparent in Lagos with a strong heterogeneity of land surface characteristics, increasing built-up and soil between the coast and mainland, but the vegetation within the city is exceedingly degraded, leading to high intensity of LST^40,41^. Thus, source landscapes are noted for large exposed concrete surfaces with high heat absorption which eventually increases the temperature in the cities while much evapotranspiration and reduction of temperature takes place in the sink landscapes.

### What are the drivers of differences in the intensity of LST?

**Figure 7:**
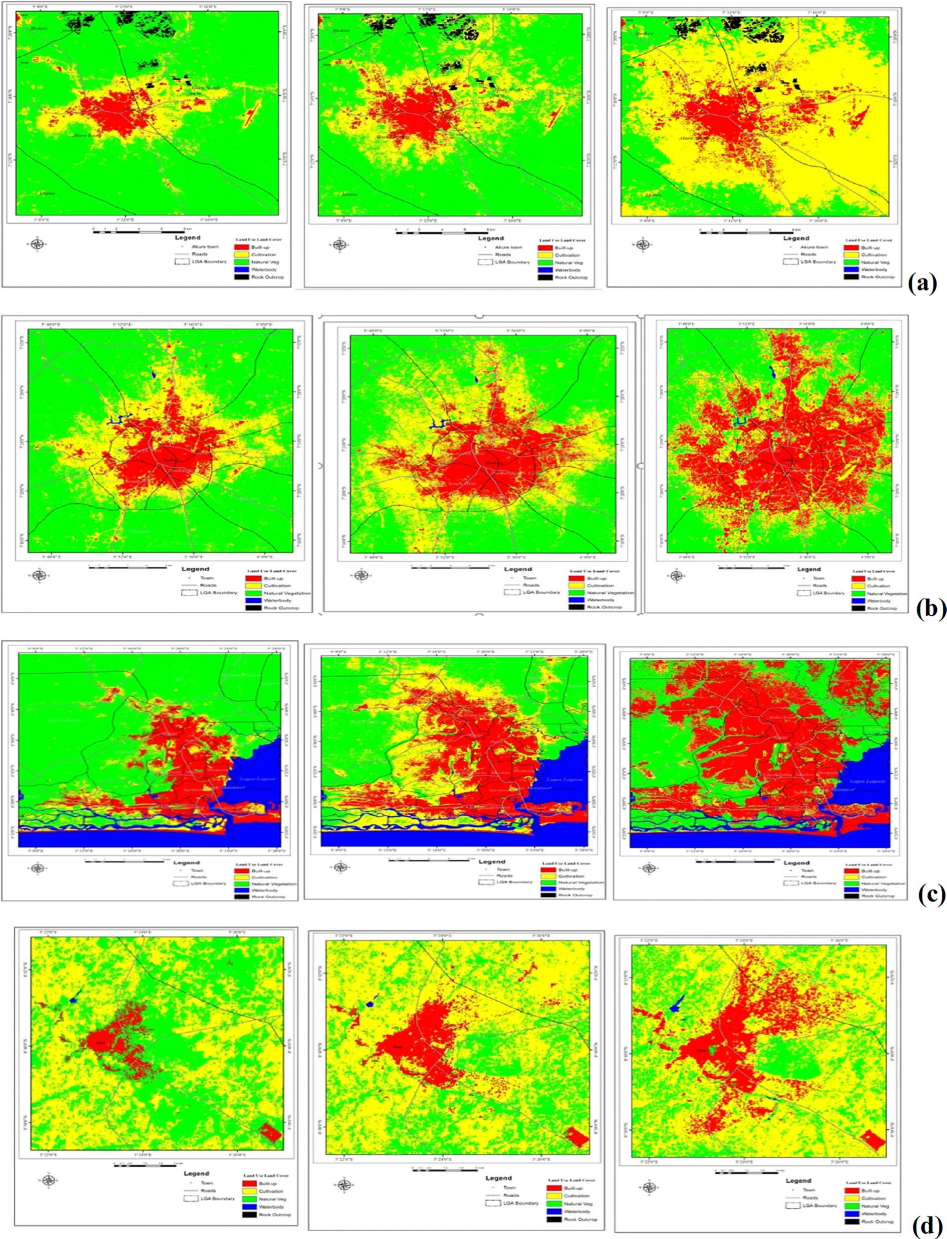
Land use/land cover classification (**a**) Akure; (**b**) Ibadan; (**c**) Lagos; and (**d**) Saki; 1984, 2004 and 2019. The Figure was generated using ArcGIS 10.5 software (http://www.esri.com/).

**Figure 8:**
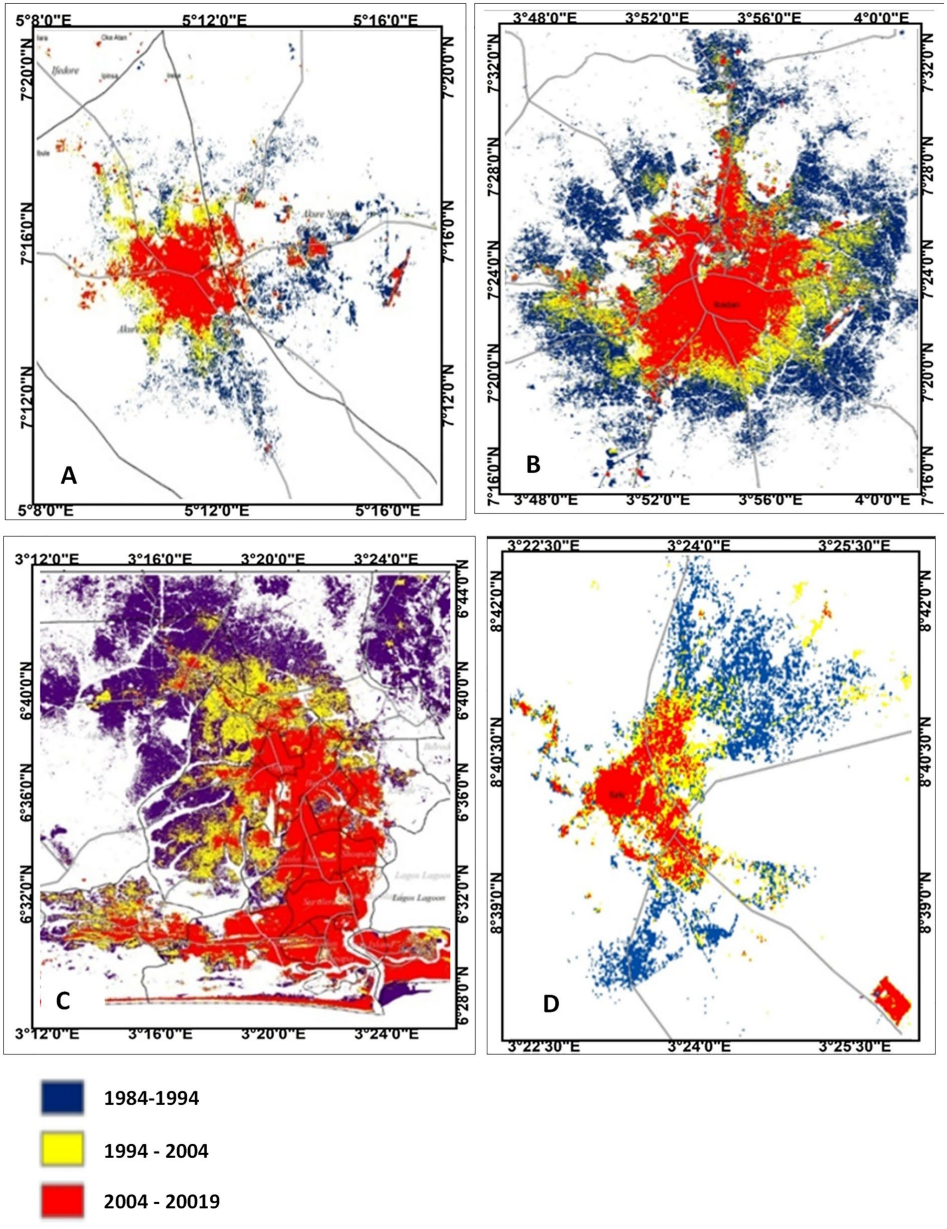
Changes in built-up area (**A**) Akure; (**B**) Ibadan; (**C**) Lagos; and (**D**) Saki. The Figure was generated using ArcGIS 10.5 software (http://www.esri.com/).

Human activities such as urban development and farm cultivation significantly altered and impacted the natural surface conditions and atmospheric properties of the urban areas. This is observed to result in different heat patterns within the cities. Changes in the LST observed in the study cities are attributed to the changes in Land use/Land cover (LULC) within and around the cities (Fig. 7, Table 7). The results reveal that as the built-up areas increases in the cities (Fig. 8), the intensity of LST is much more upsurged (Figs. 3, 4, 5, 6). Change in LULC is noticeable in all the cities examined in this study. For example, the total land area of Akure, used in this study, is approximately 1638 km^2^. In 1984, the built-up was about 33 km^2^ which increase to nearly 100 km^2^ in 2019. Cultivation also increased from 217.6 to 837.95 km^2^ in 2019. However, the vegetation decreases significantly from 1357 km^2^ in 1984 to 1067 km^2^ in 2004 and many reductions in 2019 to about 701 km^2^ (Table 7). What is obvious from these results is that as both built-up within the city and the cultivation land around the city expanding, vegetation decreases (Fig. 7a), leading to much of the LST intensification in the city (Fig. 3). A similar scenario is obvious in Ibadan in 1984 where for instance, the built-up and cultivation increase from 101 km^2^ and 242 km^2^ to 343 km^2^ and 269 km^2^ in 2019, respectively. These expansions decrease in vegetation land from 702 km^2^ in 1986 to 434 km^2^ in 2019 (Table 7).

**Table 7:** Land use/land cover change in different cities between 1984 and 2019.

LULC type	1984 (km^2^)	1994 (km^2^)	2004 (km^2^)	2019 (km^2^)
**Akure**				
Built-up	33.19	39.87	71.23	99.78
Cultivation	217.53	271.11	469.21	806.91
Natural vegetation	1357.15	1298.01	1067.45	701.22
Rock outcrop	30.16	29.02	30.03	30.01
Waterbody	0.11	0.13	0.22	0.22
Total	1638.14	1638.14	1638.14	1638.14
**Ibadan**				
Built-up	101.07	151.98	197.59	343.12
Cultivation	241.87	244.45	259.03	269.42
Natural vegetation	702.25	649.67	589.86	433.92
Waterbody	1.52	1.61	1.23	1.25
Total	1046.71	1047.71	1047.71	1047.71
**Lagos**				
Built-up	295.71	696.24	775.12	1052.51
Cultivation	193.26	235.97	581.97	164.88
Natural vegetation	1371.18	930.11	552.89	685.71
Waterbody	481.57	479.4	431.74	438.62
Total	2341.72	2341.72	2341.72	2341.72
**Saki**				
Built-up	5.98	6.99	8.91	15.98
Cultivation	72.83	74.48	76.26	71.36
Natural vegetation	42.79	40.12	36.42	34.12
Water body	0.07	0.08	0.08	0.21
Total	121.67	121.67	121.67	121.67

In Lagos in 1984, the built-up was about 296 km^2^ but increased each year to 1053 km^2^ in 2019 Cultivation, however, decreased from 193 km^2^ in 1984 to nearly 165 km^2^ in 2019. The reason for the reduction in cultivation land in Lagos is obvious, since the city is a major industrial location in the country, only a few lands are used for farming. As the built-up increases in the city, the natural vegetation in Lagos decreased from 1371.19 km^2^ in 1984 to about 553 km^2^ in 2004 and 686 km^2^ in 2019. Even the waterbody is shrinking as it reduces from 481.56 km^2^ in 1984 to about 439 km^2^ in 2019 (Table 7). Reduction in waterbody may be due to sand filling of some canals to accommodate the expansion of built-up. The total land area of Saki, used in this study, is about 122 km^2^. In 1984, the built-up was 5.98 km^2^ which increased to 8.91 km^2^ in the year 2004 and over 15 km^2^ in 2019. Natural vegetation in Saki was on constant reductions, it decreased from 42.79 km^2^ in 1984 to 34.12 km^2^ in 2019 (Table 7).

Much of the changes in the LST in the cities (Figs. 3, 4, 5, 6) are the results of changes in the built-up area (Fig. 8). The relationship between LST and LULC was further examined by calculating the zonal statistics of the images for the different years across all four cities (Fig. 6). The result reveals that the mean surface temperature variations over different land cover types. For Akure, the mean temperature over built-up ranges between 24 and 29 °C from 1984 and 2019, this is a high temperature. For the Ibadan area, the mean surface temperature variation over different land cover types revealed that the majority of the bare surface and impervious surface of Ibadan metropolis had temperatures between 22 and 38 °C, while the mean temperature for built-up ranges between 26 and 29 °C from 1984 and 2019 in which by implication of the interpretation of the zonal statistic corresponding to high temperature. For Lagos metropolis, the mean surface temperature variation over different land cover types revealed that the majority of the bare surface and impervious (built-up) surface of Lagos metropolis had temperatures between 20 and 39 °C, while the mean temperature for built-up ranges between 25 and 30 °C from 1984 and 2019. For the Saki area, the mean surface temperature variation over different land cover types revealed that the mean temperature for built-up ranges between 29 and 36 °C from 1984 and 2019 in which by implication of the interpretation of the zonal statistics corresponding to high temperature. The highest mean temperature was observed in Saki and this again is as a result of the ecological location of Saki in the Guinean Savannah.

The results are indicated that the LST is strongly correlated with change in LULC. Hence areas with the least vegetation are experiencing higher land surface temperatures. Again in this study, LST and LULC were found to be closely correlated in all land cover categories, especially in urban and vegetated/rural areas across the four cities that are being considered. Putting the side of the image by side one another could help to visually observe the relationship amongst these variables, seeing the correlation between LST and LULC in a specific location as well as their disparities as they are located in different ecological zones. Thus the results from this study reveal that anthropogenic factors such as urban encroachment into agricultural or rural land due to urban expansion and farming activities have contributed immensely to the removal of vegetation cover. As a result, this alteration in land cover is most responsible for the variation in land surface temperatures over time and space. Besides, the impervious surface is one of the most important land cover types and a feature of urban/suburban environments which affects the LST in the urban area. It is known to affect urban surface temperatures by altering the sensible and latent fluxes. Impervious surfaces represent materials that do not absorb water or dampness^40–42^. Most materials used in urban constructions, for example, housetops, roads, interstates, parking areas and walkways are impervious.

## Conclusion

The study attempted to compare spatiotemporal variations and intensity of land surface temperature (LST) in four cities located in different ecological zones. Based on the satellite dataset, remote sensing and Geographic Information System (GIS) techniques, the study assesses variations in urban LST in the cities over a period of 30 years. The drivers of LST and consequently urban heat islands, including changes in vegetation cover, bare surface (considering soil moisture content), water bodies and population (hence built density and impervious surfaces) are examined in the study. Compared to the traditional meteorological observation method, remote sensing technology has the advantage of wide coverage, which makes the large-scale assessment of LST possible^43^.

The major findings from this study reveal: (1) spatial and temporal variations in the LST in the cities; (2) change in the land use and land (LULC) are responsible for the change in the LST noted in the cities; (3) significant relationship between change LST, LULC and Ecological location of the cities. Generally, most locations in the cities with a relatively low temperature in 1984 were converted to hot spots in 2004 and hotter in 2019. What is obvious from this study is that maximum air temperature was in built-up areas of the city while the minimum temperature observed was in areas with dense vegetation. This is noticeable to be a result of an increase in built-up areas over the years and a corresponding decrease in vegetation^5,44^. This implies that the spatial and temporal changes in the land uses have greatly influenced the increase in the land surface temperature of each of the identified land uses. The study further reveals increasing variations in LST in all land uses which are results of rapid change LULC and modification of urban landscapes. Cities demonstrate greater temperature in their centre than the surrounding rural areas, which is known as Urban Heat Island effect. These forms a temperature difference between the cities and the surrounding suburbs because of the effect which causes discomfort to the city dwellers^45^. In recent years, the results from the present study demonstrate that a huge amount of vegetation cover is replaced by an artificial built surface that absorbs incoming solar radiation or heat and makes the cities warmer than ever before. The reasons for this outcome is very obvious as the majority of source landscapes in the cities centres are usually built-up surfaces distinguished from the concrete surface. According to the physical properties of the materials, thermal conductivity, and heat capacity, the materials with higher thermal conductivity conduct heat to their interior easier, whereas materials with high heat capacity store more heat and, as more heat is retained, the temperature of the material increases^40,46,47^.

It has been reported in the literature that the phenomenon exists in almost every big city. Numerous factors are held accountable for this effect, including land use and land cover changes in urban areas^48^, anthropogenic heat release, climatic conditions and air pollutants^42,45^. Lagos and Ibadan for instance are some of the fastest-growing cities in Africa today due to the rapid physical expansion of the cities. It is also reported that the population of the people living in Lagos has increased up to five times in the past 30 years which invariably means that there will be massive crowding in terms of establishing structures increased competition for amenities and increased thermal discomfort due to overcrowding of people as well as changes in land use and land cover. These findings are coherent with the results of the studies by Dewan and Corner^48^ and Ayanlade^6^ which revealed the impacts of land use and land cover changes on urban LST. Moreover, the results from the present study disclose a strong relation between LULC and urban temperature, especially regarding vegetation and built density. The major findings and innovation of this study are that different types of land cover within an urban area can affect the spatial pattern of the LST, though this varies from one ecological zone to another. Normally, the distribution of temperature in an urban area depends on its urban LULC. Based on findings from the present study, it can be established that vegetation availability plays a very low significant role in the temperature of the metropolis. The results from this study are also useful for urban heat mitigation efforts, which will aid policy maker in dealing with city challenges. Essentially as these are the factors directly related to the cities’ land surface temperature regime. The major findings from this study are useful in informing policy that will promote more sustainable urban development in the cities. What can be learned from this study is that cities’ population is expected to keep expanding and therefore there is a need to establish more viable linkages between the growing population and land use patterns, essentially because these are the factors directly related to the cities’ land surface temperature regime.

## References

[1] Masoudi M, Tan PY (2019). Multi-year comparison of the effects of spatial pattern of urban green spaces on urban land surface temperature. Landsc. Urban Plan..

[2] Zhang Y, Sun L (2019). Spatial-temporal impacts of urban land use land cover on land surface temperature: Case studies of two Canadian urban areas. Int. J. Appl. Earth Obs. Geoinf..

[3] Yang J (2020). Investigating the diversity of land surface temperature characteristics in different scale cities based on local climate zones. Urban Clim..

[4] Oke TR (1982). The energetic basis of the urban heat island. Q. J. R. Meteorol. Soc..

[5] Ayanlade A (2017). Variations in urban surface temperature: An assessment of land use change impacts over Lagos metropolis. Weather.

[6] Ayanlade A (2016). Variation in diurnal and seasonal urban land surface temperature: Landuse change impacts assessment over Lagos metropolitan city. Model. Earth Syst. Environ..

[7] Senanayake IP, Welivitiya W, Nadeeka PM (2013). Remote sensing based analysis of urban heat islands with vegetation cover in Colombo city, Sri Lanka using Landsat-7 ETM+ data. Urban Clim..

[8] Yang J (2018). Green and cool roofs’ urban heat island mitigation potential in tropical climate. Sol. Energy.

[9] Litardo J (2020). Urban Heat Island intensity and buildings’ energy needs in Duran, Ecuador: Simulation studies and proposal of mitigation strategies. Sustain. Cities Soc..

[10] Grimmond SU (2007). Urbanization and global environmental change: Local effects of urban warming. Geogr. J..

[11] Zhou X, Chen H (2018). Impact of urbanization-related land use land cover changes and urban morphology changes on the urban heat island phenomenon. Sci. Total Environ..

[12] Singh P, Kikon N, Verma P (2017). Impact of land use change and urbanization on urban heat island in Lucknow city, Central India. A remote sensing based estimate. Sustain. Cities Soc..

[13] Aghamohammadi N, Ramakreshnan L, Fong CS, Sulaiman NM (2021). Urban Heat Island, contributing factors, public responses and mitigation approaches in the tropical context of Malaysia. Urban Heat Island Mitigat..

[14] Kabano P, Lindley S, Harris A (2021). Evidence of urban heat island impacts on the vegetation growing season length in a tropical city. Landsc. Urban Plan..

[15] Stewart ID, Oke TR (2012). Local climate zones for urban temperature studies. Bull. Am. Meteorol. Soc..

[16] Rizwan AM, Dennis LY, Chunho L (2008). A review on the generation, determination and mitigation of Urban Heat Island. J. Environ. Sci..

[17] Nuruzzaman M (2015). Urban heat island: Causes, effects and mitigation measures—a review. Int. J. Environ. Monit. Anal..

[18] Weng Q, Lu D, Schubring J (2004). Estimation of land surface temperature–vegetation abundance relationship for urban heat island studies. Remote Sens. Environ..

[19] Stewart, I. & Oke, T. Newly developed “thermal climate zones” for defining and measuring urban heat island magnitude in the canopy layer. In *Eighth Symposium on Urban Environment, Phoenix, AZ* 2009 (2009).

[20] Shastri H, Barik B, Ghosh S, Venkataraman C, Sadavarte P (2017). Flip flop of day-night and summer-winter surface urban heat island intensity in India. Sci. Rep..

[21] Hu Y (2019). Comparison of surface and canopy urban heat islands within megacities of eastern China. ISPRS J. Photogramm. Remote. Sens..

[22] Ayanlade A, Jegede O (2015). Evaluation of the intensity of the daytime surface urban heat island: How can remote sensing help?. Int. J. Image Data Fusion.

[23] Sobrino JA, Li Z-L, Stoll MP, Becker F (1994). Improvements in the split-window technique for land surface temperature determination. IEEE Trans. Geosci. Remote Sens..

[24] Zhao S, Qin Q, Yang Y, Xiong Y, Qiu G (2009). Comparison of two split-window methods for retrieving land surface temperature from MODIS data. J. Earth Syst. Sci..

[25] Chatterjee RS, Singh N, Thapa S, Sharma D, Kumar D (2017). Retrieval of land surface temperature (LST) from landsat TM6 and TIRS data by single channel radiative transfer algorithm using satellite and ground-based inputs. Int. J. Appl. Earth Obs. Geoinf..

[26] Pandya MR (2014). Retrieval of land surface temperature from the Kalpana-1 VHRR data using a single-channel algorithm and its validation over western India. ISPRS J. Photogramm. Remote. Sens..

[27] Wang N (2019). Evaluation and comparison of hyperspectral temperature and emissivity separation methods influenced by sensor spectral properties. Int. J. Remote Sens..

[28] Jacob F (2017). Reassessment of the temperature-emissivity separation from multispectral thermal infrared data: Introducing the impact of vegetation canopy by simulating the cavity effect with the SAIL-Thermique model. Remote Sens. Environ..

[29] Borel, C. C. & Tuttle, R. F. In *2011 Aerospace Conference.* 1–14.

[30] Kumari B (2020). Longitudinal study of land surface temperature (LST) using mono-and split-window algorithms and its relationship with NDVI and NDBI over selected metro cities of India. Arab. J. Geosci..

[31] Abdul Athick ASM, Shankar K, Naqvi HR (2019). Data on time series analysis of land surface temperature variation in response to vegetation indices in twelve Wereda of Ethiopia using mono window, split window algorithm and spectral radiance model. Data Brief.

[32] Qin Z, Karnieli A, Berliner P (2001). A mono-window algorithm for retrieving land surface temperature from Landsat TM data and its application to the Israel-Egypt border region. Int. J. Remote Sens..

[33] Sobrino JA, Jiménez-Muñoz JC, Paolini L (2004). Land surface temperature retrieval from LANDSAT TM 5. Remote Sens. Environ..

[34] Tsou J, Zhuang J, Li Y, Zhang Y (2017). Urban heat island assessment using the Landsat 8 data: A case study in Shenzhen and Hong Kong. Urban Sci..

[35] Chander G, Markham BL, Helder DL (2009). Summary of current radiometric calibration coefficients for Landsat MSS, TM, ETM+, and EO-1 ALI sensors. Remote Sens. Environ..

[36] Ahmad A, Quegan S (2012). Analysis of maximum likelihood classification on multispectral data. Appl. Math. Sci..

[37] Otukei JR, Blaschke T (2010). Land cover change assessment using decision trees, support vector machines and maximum likelihood classification algorithms. Int. J. Appl. Earth Obs. Geoinf..

[38] Snyder WC, Wan Z, Zhang Y, Feng Y-Z (1998). Classification-based emissivity for land surface temperature measurement from space. Int. J. Remote Sens..

[39] Sobrino J, Caselles V, Becker F (1990). Significance of the remotely sensed thermal infrared measurements obtained over a citrus orchard. ISPRS J. Photogramm. Remote. Sens..

[40] Kesikoglu MH, Ozkan C, Kaynak T (2021). The impact of impervious surface, vegetation, and soil areas on land surface temperatures in a semi-arid region using Landsat satellite images enriched with Ndaisi method data. Environ. Monit. Assess..

[41] Xiao R-B (2007). Spatial pattern of impervious surfaces and their impacts on land surface temperature in Beijing, China. J. Environ. Sci..

[42] Atasoy M (2020). Assessing the impacts of land-use/land-cover change on the development of urban heat island effects. Environ. Dev. Sustain..

[43] Ayanlade A (2016). Seasonality in the daytime and night-time intensity of land surface temperature in a tropical city area. Sci. Total Environ..

[44] Das S, Angadi DP (2020). Land use-land cover (LULC) transformation and its relation with land surface temperature changes: A case study of Barrackpore Subdivision, West Bengal, India. Remote Sens. Appl. Soc. Environ..

[45] Yamamoto, Y. Measures to mitigate urban heat islands. Report No. 1349-3663, (NISTEP Science & Technology Foresight Center, 2006).

[46] Mpakairi KS, Muvengwi J (2019). Night-time lights and their influence on summer night land surface temperature in two urban cities of Zimbabwe: A geospatial perspective. Urban Clim..

[47] Dutta D, Rahman A, Paul S, Kundu A (2021). Impervious surface growth and its inter-relationship with vegetation cover and land surface temperature in peri-urban areas of Delhi. Urban Clim..

[48] Dewan AM, Corner RJ (2012). The impact of land use and land cover changes on land surface temperature in a rapidly urbanizing megacity. IEEE Int. Geosci. Remote Sens. Symp..

